# Phase II study of induction chemotherapy with TPF followed by radioimmunotherapy with Cetuximab and intensity-modulated radiotherapy (IMRT) in combination with a carbon ion boost for locally advanced tumours of the oro-, hypopharynx and larynx - TPF-C-HIT

**DOI:** 10.1186/1471-2407-11-182

**Published:** 2011-05-19

**Authors:** Alexandra D Jensen, Jürgen Krauss, Karin Potthoff, Almaz Desta, Gregor Habl, Athanasios Mavtratzas, Christine Windemuth-Kiesselbach, Jürgen Debus, Marc W Münter

**Affiliations:** 1 Dept. of Radiation Oncology INF 400 69120 Heidelberg Germany; 2 National Centre for Tumour Diseases (NCT) INF 460 69120 Heidelberg Germany; 3 Alcedis GmbH Winchester-Str. 2 35394 Gießen Germany

## Abstract

**Background:**

Long-term locoregional control in locally advanced squamous cell carcinoma of the head and neck (SCCHN) remains challenging. While recent years have seen various approaches to improve outcome by intensification of treatment schedules through introduction of novel induction and combination chemotherapy regimen and altered fractionation regimen, patient tolerance to higher treatment intensities is limited by accompanying side-effects. Combined radioimmunotherapy with cetuximab as well as modern radiotherapy techniques such as intensity-modulated radiotherapy (IMRT) and carbon ion therapy (C12) are able to limit toxicity while maintaining treatment effects. In order to achieve maximum efficacy with yet acceptable toxicity, this sequential phase II trial combines induction chemotherapy with docetaxel, cisplatin, and 5-FU (TPF) followed by radioimmunotherapy with cetuximab as IMRT plus carbon ion boost. We expect this approach to result in increased cure rates with yet manageable accompanying toxicity.

**Methods/design:**

The TPF-C-HIT trial is a prospective, mono-centric, open-label, non-randomized phase II trial evaluating efficacy and toxicity of the combined treatment with IMRT/carbon ion boost and weekly cetuximab in 50 patients with histologically proven locally advanced SCCHN following TPF induction chemotherapy. Patients receive 24 GyE carbon ions (8 fractions) and 50 Gy IMRT (2.0 Gy/fraction) in combination with weekly cetuximab throughout radiotherapy. Primary endpoint is locoregional control at 12 months, secondary endpoints are disease-free survival, progression-free survival, overall survival, acute and late radiation effects as well as any adverse events of the treatment as well as quality of life (QoL) analyses.

**Discussion:**

The primary objective of TPF-C-HIT is to evaluate efficacy and toxicity of cetuximab in combination with combined IMRT/carbon ion therapy following TPF induction in locally advanced SCCHN.

**Trial Registration:**

Clinical Trial Identifier: NCT01245985 (clinicaltrials.gov)

EudraCT number: 2009 - 016489- 10

## Background

### Radiochemotherapy

Platinum-containing radiochemotherapy is the current standard of care in the conservative treatment approach for locally advancend squamous cell carcinoma of the head and neck (SCCHN) [1]. The magnitude of survival benefit if chemotherapy was applied concomitantly with radiotherapy was 8% at 5 years as compared to radiotherapy alone in the meta-analysis undertaken by the MACH-NC Study Group [2,3]. A much smaller but still significant survival benefit was detected for all radiochemotherapy algorithms, whether applied in a neoadjuvant, adjuvant or concomitant setting with 4% at 5 years [2,3]. This small but significant survival benefit was caused mainly by an increased local control rate and only due to a small effect in reducing distant metastases. No difference in response to radiochemotherapy could be detected regarding the tumor site (oropharynx, oral cavity, larynx, and hypopharynx). In a recent update of these data, initial results could be confirmed also including more recent studies [4]. A second meta-analysis also including more recent trials in advanced SCCHN found an overall survival benefit of 12 months when adding chemotherapy to normally fractionated radiotherapy or even altered fractionation schedules [5]. A small but significant survival benefit of 3.4% can be achieved by altered fractionation schedules [6]. Hyperfractionation in particular, leads to a similar absolute improvement in overall survival (8%) as compared to radiochemotherapy [6] and accelerated-hyperfractionated radiation yielded the highest locoregional control rates in the RTOG 90-03 trial [7] though the effect of the treatment regimen on overall survival was not significant in this trial. Accelerated radiation therapy alone, especially when given as a split course radiation schedule or extremely accelerated treatments with decreased total dose, does not seem to impact overall survival. Using altered fractionation resulted additionally in an increased locoregional control rate while younger patients apparently have a higher benefit from altered fractionation schedules [6].

If choosing radiochemotherapy, the results of the MACH-NC meta-analysis indicated chemotherapy should be platinum-based [2-4]. Therefore radiochemotherapy with three cycles cisplatin 100 mg/m2 body surface given concomitantly can be considered as one of the main standards; however, this approach is associated with substantial toxicity and poor compliance.

Improvement of local control and overall survival by intensified treatment regimen comes at a price: increased toxicity of the combined approach, high number of patients unable to complete the full treatment course (between 5% [8], 15-27% [9] 37% % [10]) or treatment breaks of > 3days: 17% [8], and the still disappointing long-term results show the need to optimize these therapeutic combinations. Recent years have seen the advent of more sophisticated radiotherapy techniques, such as intensity-modulated radiotherapy (IMRT) and particle therapy, introduction of new chemotherapeutic combinations with the introduction of taxanes in the treatment of SCCHN as well as establishment of molecular targeted agents in the routine treatment of head and neck cancer.

### IMRT

Radiotherapy of head and neck cancer is commonly accompanied by marked side-effects. Modern techniques have shown to significantly reduce long-term sequelae of the treatment [11-13] and have rapidly been integrated into routine clinical practice. Techniques such as intensity-modulated radiotherapy (IMRT) and image-guided radiotherapy (IGRT) facilitate application of higher doses due to stepp gradients and hence higher conformality and improved sparing of normal tissues. Despite relative dose escalation, acute and late toxicity can be reduced. A recent phase III trial comparing IMRT versus conventional RT could clearly demonstrate a significant advantage of IMRT in reducing the rate of xerostomia [14]. In order to reduce toxicity and therefore improve radiotherapy regimens, integration of modern radiotherapy techniques into potential new trial designs is of fundamental importance.

### Particle Therapy

The physical properties of particle beams allow sharp dose gradients and hence relative dose escalation with reduction of dose to normal organs. This has been shown to improve clinical outcome especially in otherwise relatively radio-resistant tumours [15-18]. Particle therapy therefore represents another valuable tool for clinicans to achieve even higher conformality of radiation dose distributions around the tumour. In addition, heavy charged particles have shown increased relative biological effectivness (RBE) with an increase in the linear energy transfer (LET). High-LET radiation response in tissue is less influenced by oxygenation and less sensitive to variations in the cell cycle and DNA repair. Photon radiotherapy combined with heavy charged particles was established in various specalized centers in the world. Since 1997, more than 400 patients were treated with carbon ions in a cooperation between the University of Heidelberg and the GSI in Darmstadt. For malignant head and neck tumours, particle therapy at the University of Heidelberg was primarily applied for patients with adenoid cystic carcinomas in a mixed beam regimen consisting of IMRT and carbon ion boost [15,16]. In squamous cell head and neck tumours, improved outcome in recent trials has been achieved by improved local control: doses of approximately 70 Gy are needed to effecitively treat these often centrally necrotic tumours in a definitive setting. Relative dose escalation, increased biological effectiveness and oxygen independence of radiation effect therefore provide a rationale for the evaluation of particle therapy in the multi-modality treatment of advanced SCCHN.

### EGF-Receptor Inhibition and Radiotherapy

A quantum leap of radiation oncology in SCCHN has been the establishment of combined radioimmunotherapy with the monocloncal EGFR-antibody cetuximab for locally advanced tumors of the head and neck [19,20].

The EGF-receptor is over expressed at high levels in nearly all squamous cell carcinomas of the head and neck region. An increased expression of the EGF-receptor is associated with a poor prognosis [21].

The "Bonner" phase III trial compared radiotherapy with cetuximab with radiotherapy alone for definitive treatment. In this study, cetuximab was shown to significantly improve both overall survival and local control. Apart from skin reactions and a slight increase of infusion reactions no further severe side effects were reported. In contrast to combined radiochemotherapy regimen, combination of radiotherapy with cetuximab did not increase radiotherapy-associated side-effects such as mucositis [19]. This was also the first study to demonstrate in a clinical phase III setting that the combination of radiotherapy and a monoclonal antibody against the EGF-receptor resulted in a clear benefit for this combination. Retrospective comparison of the data with prior radiochemotherapy studies showed comparable clinical results without the sometimes marked side effects of the chemotherapy approach [22].

In vitro studies demonstrated that treatment with Cetuximab inhibited ligand-induced EGFR autophosphorylation and EGFR-dependent cellular response, including extracellular acidification, cell proliferation, and production of angiogenic factors by tumour cells [23]. Cetuximab alone (monotherapy) eradicated established xenograft A431 epidermoid carcinoma tumours and inhibited tumour growth of breast, renal, pancreatic, head and neck, prostate, ovarian, and non-small cell lung carcinomas (NSCLC; including NSCLC xenograft tumors that express clinically relevant kinase domain mutants) that expressed 9000 to greater than1.6 × 10^6 ^EGFR molecules/cell [24]. Cetuximab is being studied as a monotherapy and in combination with radiotherapy and/or chemotherapy in several clinical studies. Efficacy has been observed in various tumour types if given as monotherapy or in combination with chemotherapy. Combination therapy of Cetuximab and chemo-therapeutic agents resulted in greater inhibition of tumour growth in colon, lung, breast, ovarian, pancreas, and head and neck xenograft tumors than either agent alone [25]. The results of a direct comparison between radiotherapy and antibody versus radiochemotherapy evaluated in the current RTOG 0522 protocol, initial results are currently pending but expected for 2011.

### Induction chemotherapy for SCCHN

A recently established conservative treatment concept in SCCHN is the use of induction chemotherapy followed by radiotherapy or radiochemotherapy. Two fully published phase III trials investigated the antitumor activity and toxicity of two induction chemotherapy regimens of docetaxel, cisplatin and fluorouracil (TPF) versus standard cisplatin and 5-FU (PF) [26-28]. Both of these trials showed a significantly improved overall survival in the experimental (TPF) arm. In the trial published by Vermorken et al. [27] induction chemotherapy was followed by radiotherapy only. The second trial presented by Posner et al. [26] induction chemotherapy was followed by chemoradiotherapy. However, one of the limitations of this trial were comparatively conservative radiation doses employed. So far, no phase III trial has yet published results of a direct comparison between sequential TPF induction followed by radiotherapy/chemoradiation and radiochemotherapy without induction. Preliminary data presented at ASCO 2009 [29] for the first time showed a benefit in overall survival in favour of the sequential regimen. Toxicity was only slightly increased by the addition of taxanes in all the presented trials. Nevertheless, a full course - usually consisting of three cycles - induction chemotherapy with TPF - followed by a full course of radiochemotherapy is a very intensive treatment concept, few of the patients are able to tolerate. Only a limited number of patients can be treated according to protocol. Several phase III trials arer currently addressing this issue and are designed to investigated if efficacy can be maintained if radiotherapy in the sequential approach is combined with cetuximab in order to reduce toxicity. As explained above, combined raidioimmunotherapy with cetuximab is able to achieve comparable control rates as compared to combined radiochemotherapy in the primary treatment setting [19,20].

The TPF-C-HIT trial is a phase II trial evaluating radioimmunotherapy with cetuximab and radiation as intensity-modulated radiotherapy with carbon ion boost following TPF induction.

## Methods/design

### Study design

The TPF-C-HIT trial is a prospective, non-randomized phase II feasibility trial evaluating locoregional control (LRC) of radioimmunotherapy with cetuximab as carbon ion boost and IMRT following standard induction chemotherapy with TPF as primary endpoint. Secondary endpoints are disease-free survival (DFS), progression-free survival (PFS), overall survival (OS), acute/late effects as well as any adverse events of radioimmunotherapy as carbon ion boost and IMRT.

### Study Characteristics

As explained above, long-term local control in advanced squamous cell head and neck cancer (SCCHN) remains a therapeutic challenge. In order to further improve local control for these patients after standard induction chemotherapy with TPF, raster-scanned carbon ion therapy, and radioimmunotherapy as IMRT as the so far most effective treatment components are combined in order to achieve the best possible results. This combination regimen will be tested as to efficacy and toxicity profile. TPF-C-HIT is a monocentric, open-label, non-randomised phase II trial.

**Figure 1:**
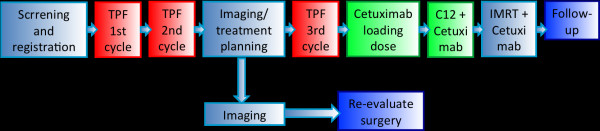
**trial flow-chart**.

Patients on the trial receive 2 cycles of induction chemotherapy with docetaxel, cisplatin, and 5-FU followed by re-staging imaging. In case of treatment response, these patients receive their radiation therapy planning scan and one further cycle of TPF. In case of tumour progression after 2 cycles of TPF, according to international recommendation and guidelines, surgery should be re-evaluated as salvage treatment. After completion of the third course of TPF, patients go on to combined radioimmunotherapy: 8 fractions of carbon ion therapy at 3 GyE per fraction (total dose 24 GyE) to the primary tumour and involved lymph nodes are followed by IMRT to the whole neck at daily fractions of 2 Gy up to a total dose of 50 Gy. Cetuximab loading dose is given 7 days prior to commencement of radiotherapy followed by weekly doses throughout the treatment. Treatment duration will therefore be 108 days (corresponding to approximately 16 weeks). An overview of the trial schedule is provided in figure 1 (Figure 1). The trial will also be supervised by an independent data monitoring committee consisting of two radiation oncologists in accordance with the EMEA guidelines on data monitoring committees.

### Study objectives

To evaluate efficacy and toxicity of radioimmunotherapy with cetuximab as carbon ion boost and IMRT following standard induction chemotherapy with TPF. Primary endpoint is local control at 12 months post completion of treatment. Secondary endpoints are disease-free survival, progression-free survival, overall survival, acute and late radiation effects as well as any adverse events according to CTCAE v. 4.0, and proteomic and genomic analyses. For development of prognostic markers, proteomic and genomic analyses are also included in the secondary endpoints.

### Sample size/number of subjects

The main analysis is based on the hypothesis that an absolute increase of local-regional control rate at 1 year of 17% is considered to qualify the study treatment as very promising for additional testing. The decision rules declared by this design with the above characteristics are as follows:

H_0_: p ≤ p_0 _= 63% vs. H_1_: p ≥ p_1 _= 80%.

p_0 _= 63% is the clinically "uninteresting" local-regional control rate at 1 year, corresponding to the null hypothesis H_0_(according to [19]).

p_1 _= 80% is the clinically "interesting" local-regional control rate at 1 year, corresponding to the alternative hypothesis H_1_.

Sample size calculation for the TPF-C-HIT trial is based on a Fleming's single stage design for phase II studies. With a significance level of 0.05, a power of 80%, π_0 _= 0.63 and π_1 _= 0.8, a sample size of 45 patients was calculated. Assuming a drop out rate of 10%, 50 need to be recruited for the trial.

#### Efficacy parameters

Locoregional control (LRC) will be assessed according to the RECIST criteria [30] with the loss of locoregional control defined as the first evidence of local progression or to death due to any cause. Local progression is defined as a progressive disease (PD) according to RECIST. The smallest measurement recorded since treatment start (nadir) will serve as a reference for further assessments of locoregional control.

Disease-free survival, progression-free survival, and overall survival are calculated from treatment start (corresponding to day 1 of induction chemotherapy) to first event (i.e. evidence of loco-regional failure, distant failure, second primary tumor, or death due to any cause. If the patient has no evidence of the before mentioned events, survival is censored at the time of last documented evaluation of efficacy/contact. Survival parameters will be calculated using the Kaplan-Meier estimation method [31]. All patients who have received cetuximab at least once will be included in the analysis on an intent-to-treat basis.

#### Safety parameters

Adverse events are defined as any untoward medical occurrence in a patient from the beginning of radioimmunotherapy (day 57) up to the follow-up visit 12 months post completion of combined radioimmunotherapy. All adverse events under standard induction chemotherapy and prior to the first administration of cetuximab will be recorded as medical history. Adverse Events will be reported using NCI CTCAE v4.0.

## Patient selection

### Inclusion criteria

• Histologically confirmed locally advanced (stage III or IV), non-metastatic squamous cell carcinoma of oro-, hypopharynx and larynx (T2-4, any N, M0),

• Oral cavity or oro-, hypopharynx or laynx as the primary tumor site,

• At least one uni-measurable lesion according to the RECIST,

• Karnofsky performance Status > 70%,

• Adequate bone marrow function: neutrophils > 1.5 × 10^9^/l, platelets > 100 × 10^9^/l, hemoglobin > 10.0 g/dl,

• Adequate liver function: Bilirubin < 1.5 mg/dL, SGOT, SGPT < 3 × ULN, GGT < 5 × ULN,

• Adequate renal function: GFR> 70 ml/min.,

• Age between 18 to 70 years,

• Life expectancy of at least 6 months,

• Ability of subject to understand character and individual consequences of clinical trial,

• Signed written informed consent,

• Negative serum/urine Beta-HCG test in women of childbearing potential,

• effective contraception for patients in procreative age

### Exclusion criteria

• Previous systemic chemotherapy, radiotherapy or surgery for carcinoma of the head and neck or and larynx,

• Nasopharyngeal carcinoma,

• M1 (distant metastases)

• Prior exposure to EGFR pathway targeting therapy,

• Participation in other interventional trials within the last 30 days,

• Any surgery within the last 30 days,

• Known allergic or hypersensitivity reaction to any drugs scheduled for the study treatment,

• signs of active infection

• Symptomatic peripheral neuropathy CTC grade 2 or higher,

• ototoxicity CTC grade 2 or higher, except if due to trauma caused by tumour mass,

• other serious illnesses or medical conditions: therapy-refractory unstable heart disease, congestive heart failure NYHA °III and °IV; coagulopathies

• Other previous malignancy within the past 5 years except prior, adequately treated basal cell carcinoma of the skin or pre-invasive carcinoma of the cervix

• Significant neurological or psychiatric condition including dementia or seizures or other serious medical condition prohibiting the patient's participation in the trial by judgement of the investigators

• Legal incapacity or limited legal capacity

• Positive serum/urine β-HCG/pregnancy

• Drug abuse

## Study treatment

### Induction chemotherapy

Patients receive three cycles of docetaxel/cisplatin/5-FU (TPF) according to the schedule described by Vermorken et al [27] at the following doses:

Docetaxel 75 mg/m² body surface (d1, d22, d43)

Cisplatin 75 mg/m² body surface (d1, d22, d43)

5-FU 750 mg/m² body surface (d1-5, d22-26, d47-51)

As a standard, patients of course receive adequate premedication with corticosteroids (dexamethsone) prior to application of docetaxel, antiemesis, and hydration according to standard recommendations of the vendours. In addition, patients receive prophylactic antibiotic treatment with ciprofloxacine 500 mg po bid for 10 days starting one week after commencement of each cycle. Dose reductions will be performed as recommended by the vendour.

Pegylated G-CSF is administered prophylactically if a prior episode of febrile neutropenia or infection with either delayed recovery of absolute neutrophil count at day 21 or grade 4 neutropenia (ANC < 0.5 × 10^9^/l) persisting for ≥ 7 days was observed during cycle one or two of TPF.

### Immunotherapy

Cetuximab is administered as 400 mg/m² body surface loading dose 7 days prior to RT-treatment start (d57) after administration of anti-histamines (dimetindene) and corticosteroids (dexamethasone).

Weekly administrations of Cetuximab 250 mg/m2 body surface follow for the duration of radiotherapy from d64.

## Radiotherapy

### Immobilisation/planning examinations

Patients are immobilized using individual thermoplastic head masks with thermoplastic shoulder fixation. Planning examinations consist of a planning CT scan (3 mm slice thickness) with the patient positioned in the individual fixation device and contrast-enhanced MRI for 3D image correlation.

### Target volumes

Gross Tumor Volume (GTV) includes the gross primary tumour or involved node(s) based on clinical and endoscopic examinations, CT scan, or other imaging techniques if applicable. Involved lymph nodes are defined as any lymph nodes > 1 cm or nodes with a necrotic center.

The Clinical Target Volume (CTV) is defined as GTV plus surrounding areas at risk for containing microscopic disease as delineated by the attending radiation oncologist. CTV1 includes the GTV with a margin of approximately 0.2 cm. CTV2 represents the nodal levels to receive elective irradiation. The CTV margins may be smaller if the GTV is adjacent to the spinal cord or other critical normal tissues. Lymph node levels are delineated in accordance with current guidelines and recommendations [32-36] as follows:

• Submental nodes (surgical level IA): In cases where the floor of mouth or level IB are involved.

• Submandibular nodes (surgical level IB): All cases except primary palate tumours which do not extend to the tonsil or base of tongue. Only the ipsilateral level IB is included unless the primary tumour reaches or crosses the midline. The ipsilateral level IB must be included in all cases if upper jugular metastases are found.

• Upper deep jugular (junctional, parapharyngeal) nodes: all cases (at the neck side ipsilateral to the primary tumour).

• Subdigastric (jugulodigastric) nodes, midjugular, lower neck, and supraclavicular nodes (levels II through IV): all cases, bilaterally, except for tonsillar fossa tumours > 1 cm from from the midline and surgical node negative contralateral neck.

• Posterior cervical nodes (level V): all cases, at the neck side where there is evidence of jugular nodal metastases.

• Retropharyngeal nodes: all cases.

The *Planning Target Volume (PTV1 and PTV2)*includes CTV1 and CTV2 with an additional margin in order to compensate for set-up variability and internal organ motion. A minimum margin of 0.2 cm around the CTV in all directions is required to define each respective PTV, except for situations in which the CTV is adjacent to spinal cord or other critical normal tissues. In such situations, the margin can be reduced judiciously if image guidance is used regularly.

## Planning and RT treatment technique

### Carbon ion therapy

Carbon ion therapy treatment planning is carried out using a dedicated treatment planning system (TPS) developed for and in co-operation with HIT (Heidelberg ion beam therapy centre). TPS offers the following functionalities also expected in conventional radiation therapy as well as methods for biological RT treatment optimization. As ion beams exhibit an increased biological effective dose depending on various factors, these need to be included within the planning algorithm. In addition, steering parameters for scanned ion beams need also be calculated by the TPS.

Carbon ion treatment is given at the HIT after inverse treatment planning in active beam application (raster-scanning method). A monoenergetic ion beam with a full-width/half-maximum (FWHM) of 5 mm is extracted from the accelerator system (synchrotron) and magnetically deflected to subsequently scan all planned iso-energetic slices roughly corresponding to the tumour's radiological depth. Using this method almost any desired dose distribution can be created.

In view of the tolerance dose to organs at risk a dose of 24 GyE in 8 fractions for carbon ions will be prescribed to the median of the calculated dose distribution for the target volume (PTV1). Dose specification is based on biologic equivalent dose because of the high relative biologic effectiveness (RBE) of carbon ions Treatment planning aims at covering the PTV1 with the 95%- and 90%-treatment isodose.

Our policy is to avoid under-dosage within a target volume if the dose constrains for the normal tissue are not exceeded.

### Intensity-modulated radiotherapy (IMRT)

Radiotherapy will be given to the patients only as intensity modulated radiotherapy (IMRT) and must be used for the entire course of treatment. Intensity-modulated RT is planned using two commercially available planning systems: KonRad (Siemens OS) for step-and-shoot applications or Tomotherapy^®^. IMRT hence is delivered either at a 6 MV-linear accelerator (step-and-shoot technique) or at a 6 MV tomotherapy unit to a total dose of 50 Gy in 2 Gy/fraction. IMRT image guidance consists of MV cone-beam CTs which are taken into account for the calculation of the total applied dose.

### Dose Prescription and plan evaluation

IMRT will be given in 25 fractions over approximately 5 weeks. The primary tumour, involved nodes (PTV1) and subclinical disease sites (PTV2) will receive 2.0 Gy per day. The total doses for the PTV1 and the PTV2 will thus be 50.0 Gy± 5%, respectively. Treatment breaks should be minimized, any break of more than 5 days will be considered a major protocol violation.

All plans must be normalized such that 95% of the volume of the PTV1 is covered with the prescribed dose. In addition the following requirements must be met:

• No more than 20% of the PTV1 should receive ≥ 110% of the prescribed dose.

• No more than 5% of PTV1 or PTV2 should receive ≤ 90% of the prescribed dose.

• No more than 2% or 2 cc of tissue outside the PTVs should receive ≥ 110% of the prescribed dose to the PTV1.

### Critical normal structures constraints

The constraints are applicable for the summation (carbon ion and photon IMRT) plan.

• Spinal cord: a margin of 0.5-1 cm around the spinal cord may be added to create a Planning Organ at Risk Volume (PRV). The dose to any point within the spinal cord should not exceed 45 Gy to any volume larger than 0.03 cc.

• Parotid glands: the objective is to limit the mean dose to at least one gland to ≤ 26 Gy; alternatively at least 20 cc of the combined volume of both parotid glands to < 20 Gy or at least 50% of one gland to <30 Gy.

• Glottic larynx: the dose to the larynx should be kept <45 Gy whenever feasible.

• Brachial plexus: the dose to the brachial plexus must be limited to ≤ 60 Gy in patients with suspicious level IV node(s).

• Mandible: 70 Gy should not be exceeded at any point.

• Brain stem: the tolerated dose is 54 Gy, in volumes of appox. 1 cc the dose can be 60 Gy.

• Unspecified tissue outside the target volumes: ≤ 100% of the dose prescribed to PTV2. No more than 5% of the non-target tissue can receive greater than the dose to PTV2.

### Image guidance (IGRT)

Portal image of each field or orthogonal images that localize the isocenter placement of IMRT must be obtained on the first day of therapy. At least weekly set-up controls using MV cone-beam CTs (cb-CTs) are required for the IMRT treatment, daily IGRT is required for carbon ion therapy. Doses applied to the patient by the acquisition of position controls may be taken into account for calculation of overall applied dose.

### Planning objectives

Parotid glands:

1. Mean dose to either parotid < 26 Gy or

2. At least 50% of the either parotid gland will receive < 30 Gy or

3. At least 20 ml of the combined volume of both parotid glands will receive < 20 Gy.

Submandibular/sublingual glands and oral cavity: Reduce the dose as much as possible.

### Planning priorities

Critical normal structure constraints followed by the prescription goals are the most important planning priorities. The priorities in addressing the protocol aims and constraints will be in the following order:

1) Critical Normal Structure Constraints,

2) Prescription Goals,

3) Planning Goals: salivary glands.

## Treatment schedule/follow-up

### Treatment schedule

After inclusion into the trial and the patient's written informed conset, the patient receives two cycles of TPF followed by imaging for re-staging. Without evidence of progressive disease (PD) according to RECIST, the patient then receives an individual fixation device consisting of thermoplastic head mask and shoulder fixation as well as RT treatment planning scans (CT/MRI). Subsequently, patients are administered the third cycle of TPF (Figure 1). Cetuximab loading dose is applied on day 57 (7 days prior to RT start). On day 64 (RT wk 1), the patient receives the first weekly Cetuximab (250 mg/m² body surface) as well as the first fraction of carbon ion therapy (Figure 1). First, 8 fractions of C12 heavy ion boost to the PTV1 to a total dose of 24 GyE at 3 GyE per fraction (5 fractions per week) are applied. Subsequently, the patient receives intensitiy-modulated photon radiation therapy (IMRT) to the PTV2 to a dose of 50 Gy at 2 Gy per fraction (5 fractions per week). Overall, the total dose adds up to 74 GyE in 33 fractions.

**Figure 2:**
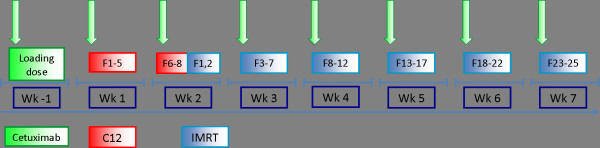
**combined radioimmunotherapy schedule**.

Cetuximab is given weekly throughout the course of radiotherapy (Figure 2).

In case of progressive disease (PD) on the re-staging examination after two cycles of TPF, surgical salvage should be re-evaluated.

### Follow-up

First follow-up examination including diagnostic, contrast-enhanced MRI will be carried out 6 weeks post completion of radiation treatment. Further controls including MRI are 3, 6, and 12 months thereafter, in 6 monthly intervals until 2 years post RT, then in yearly intervals (Figure 2). Abdominal ultrasound will be carried out q6months, chest-CT q12months. At each follow-up appointment, patients receive a symptom-oriented clinical examination, also patients' performance state (Karnofsky-Index), therapy-associated side effects as well as potential intercurrent therapy is recorded.

Patients also undergo regular check-ups incl. full ENT clinical examinations at least every 12 weeks for the first year of follow-up. Quality of life will be assessed using the EORTC head and neck questionnaire in 6-monthly intervals up to 5 years.

### Trial duration

The trial for the individual patient is completed after an 18-months follow-up period. In order to include 50 patients, an accrual period of 2 years is expected.

### Assessment of efficacy

Assessment of efficacy will be carried out by evaluation of imaging studies (CT/MRI) at each follow-up. Tumour response will be evaluated according to the RECIST-criteria [30].

### Proteomics and Genomics

If the patient has given (separate) informed consent, 30 mL venous blood will be collected from each subject for the proteomic examinations prior to the first administration of TPF, (e.g. on day -7/day 1), at day 50 (before the first treatment with cetuximab), day 64, day 73 and 106 of the treatment phase and once at the end of therapy visit (day 108). Thus, the overall volume of blood samples used for Proteomic/Genomic investigations will be approximately 120 ml. Following parameters/pathways will be investigated:

• In order to predict the efficacy of the trimodal therapy blood will be collceted during therapy and follow-up to detect and correlate the levels of well known tumor- and angiogenesis markers (VEGF, TGF-Alpha, bFGF, IL8, k-ras, etc.) using Enzyme-Linked Immunosorbent Assay (ELISA). Further, platelet protein content (i.e. tumor angiogenesis growth factors and cytokines) will be analyzed using citrate blood samples and correlated with serum- and plasma- protein results.

• In order to perform the genomic analysis, patients' blood samples are collected as indicated and RNA, miRNA and DNA isolation will be performed. Based on an established platform, linear RNA-amplification, labelling and hybridization on human genome wide oligo-arrays (transcriptome analysis) are planned. DNA samples are used to identify potential chromosomal aberrations or epigenetic alterations that might predict treatment response. RNA and miRNA samples are further analyzed by real time quantitative RT-PCR to confirm microarray data and to test a subset of clinical predictors.

The determinations of proteomic and genomic parameters will be carried out at the Department of Radiation Oncology in Heidelberg.

No further genetic investigations on the blood collected during the study will be carried out!

### Trial organization/coordination

The TPF-C-HIT trial has been designed by the Department of Radiation Oncology, and is carried out at the National Centre for Tumour disease (NCT) Heidelberg, the Heidelberg Ion Therapy Centre (HIT), and the department of Radiation Oncology, University of Heidelberg. It is an investigator-initiated trial; the Department of Radiation Oncology is responsible for co-ordination, overall trial management, registration (clinicaltrials.gov Identifier: NCT 01245985); EudraCT registration (EudraCT number: 2009 - 016489- 10), database management, quality assurance, monitoring, and reporting is carried out by Alcedis GmbH, Gießen, Germany.

### Investigators

Patients are recruited by the Department of Radiation Oncology, Heidelberg, Germany. Induction chemotherapy with TPF is given and monitored by the NCT, Heidelberg, Germany, combined radioimmunotherapy is carried out by the Department of Radiation Oncology/HIT, Heidelberg, Germany.

### Adverse events

Adverse and serious adverse events are recorded using NCI common toxicity criteria for adverse events (CTCAE v. 4). Acute radiation effects are defined as effects occurring within 90 days from beginning of radiotherapy. Late effects are defined as effects observed thereafter. Safety analysis is performed with respect to frequency of serious adverse events and adverse events stratified by organ system, severity, causality.

### Regular completion of the trial

Patient accrual is completed with inclusion of the last patient and should extend for approximately 2 years from trial initiation. Regular trial participation for each patient terminates 18 months from first administration of TPF or the patient's death respectively.

### Discontinuation of treatment

• Patient wish

• Cetuximab treatment delay for more than 2 consecutive weeks,

• Occurrence of any grade 4 toxicities related to cetuximab,

• Occurrence of >/= grade 3 allergic/hypersensitivity reaction related to cetuximab.

• Medical condition necessitating treatment termination and withdrawal of the patient from the trial

• Pregnancy

• Lack of compliance

### Premature termination of the trial

The trial can be prematurely closed or suspended by the principal investigator (PI) in following cases:

• Medical or ethical reasons relevantly affecting the risk-benefit relationship,

• Difficulties in recruitment of subjects suggest unjustifiable prolongation of the study timeline,

• Previously unexpected adverse events (in respect of their nature, severity, duration or outcome) occur with unjustifiable frequency,

• Expected adverse events occur with an unexpectedly high incidence,

• Relevant superiority of patients in one treatment arm of a comparable clinical trial,

• Legal authorities' decision

The Ethics Committee (EC) and the competent regulatory authorities will be informed about premature closure of the trial. Furthermore, the Ethics Committee(s) and competent regulatory authorities themselves may decide to stop or suspend the trial.

If the trial is closed prematurely, the trial material such as completed, partially completed, and blank CRFs will be returned to the coordinating investigator.

All involved investigators have to be informed immediately about a cessation or suspension of the trial. The decision is binding on all investigators.

### Ethics, informed consent, and safety

The final protocol was approved by the University of Heidelberg Medical School ethics committee (AFmo-167/2010), the German radiation protection authority (Bundesamt für Strahlenschutz: = BfS) and Paul-Ehrlich Institute (: = PEI). The trial complies with the Helsinki Declaration in its recent German version, the Medical Association's professional code of conduct, principles of Good Clinical Practice (GCP) guidelines and the Federal Data Protection Act. It will be carried out in keeping with local legal and regulatory requirements. Medical confidentiality and Federal Data Protection Act will be followed. Written informed consent is obtained from each patient in oral and written form.

## Discussion

With the introduction of novel radiotherapy techniques such as IMRT and particle therapy (neutron and carbon ion therapy), higher local control rates in radioresistant tumours such as adenoid cystic carcinoma could be achieved over the last decade [15,16]. Taxane-containing induction chemotherapy was shown to improve both response rates and overall survival in advanced SCCHN [26-28]. On the other hand, novel targeted agents such as the EGFR antibody cetuximab produced control rates comparable to standard radiochemotherapy regimens in retrospective comparisons with a considerably more favourable toxicity profile [19,20,22].

With the available data, there are various options to intensify treatment for patients with locally advanced head and neck tumours: modern radiotherapy techniques provide tools for relative dose escalation within the tumour, particle therapy may further improve local control by increased biological effectiveness, and induction chemotherapy improves long-term survival in these patients. All of these treatment approaches have shown clinical feasibility and efficacy as single modalities.

Current standard practice in Europe mostly consists of combined radiochemotherapy in the conservative management of locally advanced head and neck tumours (SCCHN). Management of acute treatment side-effects in clinical routine shows further intensification is rarely possible albeit feasible in terms of improved outcome. Assuming toxicity of combined radioimmunotherapy being less severe than in radiochemotherapy as demonstrated by Bonner and co-workers [19], this may still be feasible following TPF induction.

The TPF-C-HIT trial is a phase II trial to evaluate three modern treatment algorithms for head and neck cancer: Patients receive TPF induction chemotherapy followed by radioimmunotherapy with cetuximab as intensity-modulated radiotherapy (C12) plus carbon ion boost. IMRT is applied in conventional doses per fraction and total dose. The primary tumour and involved lymph nodes will be boosted by application of a carbon ion boost in active beam application (raster-scanning method). Due to these highly sophisticated RT techniques no increased risk of side effects associated with radiotherapy is expected as compared to conventional techniques and may even reduce potential side effects in this setting. Due to the advantages of IMRT and carbon ion therapy in receiving a better dose distribution in the target volume improved local control rates may be achieved. To our knowledge, this is the first clinical trial evaluation this trimodal treatment regimen.

## Conflicts of interests

Prof. Debus is a member of the Merck KGaA advisory board.

## Authors' contributions

All authors have read and approved the final manuscript.

MWM, JK, ADJ, and JD developed the study protocol and planned the trial. AH is responsible for statistical considerations/basis of the trial. All authors are responsible for conducting and co-ordination of the trial as well as patient recruitement. All authors read and approved the final manuscript

## Pre-publication history

The pre-publication history for this paper can be accessed here:

http://www.biomedcentral.com/1471-2407/11/182/prepub
